# Assessment and Decision-Making in Complex Limb Fracture Management

**DOI:** 10.7759/cureus.83877

**Published:** 2025-05-11

**Authors:** Daniel I Koshy, David Koshy, Zabrang Ishaku

**Affiliations:** 1 Trauma and Orthopaedics, Royal London Hospital, London, GBR; 2 Orthopaedics, University Hospital Plymouth, Plymouth, GBR; 3 Orthopaedics, St John of God Midland Public and Private Hospitals, Perth, AUS; 4 Trauma and Orthopaedics, Barts Health NHS Trust, London, GBR

**Keywords:** complex fractures, complex lower limb trauma, limb salvage, orthopaedics, trauma

## Abstract

Complex limb fractures, particularly those resulting from high-energy trauma, pose significant challenges in orthopaedic trauma care due to the severity of bone and soft tissue damage, risk of infection, and the need for coordinated multidisciplinary management. This narrative review explores the comprehensive assessment and decision-making process involved in managing these injuries, including clinical evaluation, radiological imaging, and functional considerations. It critically examines factors influencing the choice between limb salvage and primary amputation, such as haemodynamic stability, injury severity, predictive scoring systems like the Mangled Extremity Severity Score (MESS), infection risk, functional outcomes, psychological well-being, and economic implications. Although limb salvage offers the potential to retain the native limb, it is often associated with prolonged recovery, multiple surgical interventions, and higher complication rates. Conversely, amputation may enable a more predictable and expedited return to function, particularly when extensive soft tissue loss or neurovascular compromise is present. The review emphasises that no single scoring system or algorithm can fully dictate management decisions, which must be individualised and guided by both clinical judgment and patient-centred goals. A multidisciplinary approach involving orthopaedic, vascular, and plastic surgeons, along with rehabilitation and mental health support, is essential to optimise outcomes. Future directions should focus on improving predictive tools and rehabilitation strategies to support evidence-based, holistic decision-making in complex limb trauma.

## Introduction and background

Complex limb fractures, particularly those resulting from high-energy trauma, present a significant challenge in orthopaedic trauma care due to their severity, potential complications, and the need for multidisciplinary management. Accurate assessment is crucial for determining the appropriate intervention to optimise functional recovery, minimise long-term disability, and decide whether to pursue limb salvage or opt for primary amputation. The choice depends on multiple variables, including the severity of soft tissue damage, vascular injury, patient comorbidities, infection risk, functional outcomes, and psychological well-being. This article critically evaluates the assessment of complex limb fractures, considering clinical, radiological, and functional parameters while incorporating evidence from the literature. It also examines the factors influencing the decision between salvage and amputation in complex limb fractures, utilising published evidence to support the arguments [1,2].

## Review

Defining complex limb fractures

Complex limb fractures are defined or characterised by extensive bone and soft tissue damage, often involving comminution, displacement, or open fractures [1]. These injuries are commonly caused by high-energy trauma. Some examples would be road traffic accidents, falls from height, and crush injuries [2].

Assessment 

Clinical Assessment

A systematic clinical assessment is key in complex fracture evaluation. The Advanced Trauma Life Support (ATLS) guidelines advocate a primary survey addressing life-threatening conditions before secondary assessments [3]. The secondary survey focuses on limb-specific evaluation, including deformity, neurovascular status, soft tissue integrity, and compartment syndrome.

Deformity: When a limb deformity is noted, there should be a high index of suspicion for fracture. Further clinical and radiological assessments should be undertaken.

Neurovascular status: According to ATLS guidelines, limbs are evaluated in terms of motor function, sensation, and circulation. The presence of vascular compromise or nerve injury significantly influences management strategies. In cases where vascular damage is suspected, further imaging should be considered.

Soft tissue integrity: Soft tissue integrity is particularly important in complex fractures. The soft tissue overlying the fracture site must be thoroughly examined, as the presence of an open fracture alters the management plan. Open fractures are classified using the Gustilo-Anderson system. The British Orthopaedic Association (BOA) has released guidelines on the management of open fractures, recommending administration of antibiotics within the first hour, appropriate imaging, documentation of neurovascular status, and splinting and realignment of the limb. The timing of surgical intervention is based on the degree of contamination, as outlined in these guidelines [4].

Compartment syndrome assessment: The BOA has also provided guidance on the assessment and management of compartment syndrome. Clinical indicators such as pain out of proportion, pain on passive stretch, pallor, paraesthesia, and pulselessness are key in making the diagnosis. In patients with significant injuries or who are obtunded, intracompartmental pressure monitoring can be considered [5].

Radiological Assessment

Radiographic evaluation plays a vital role in diagnosing complex fractures, guiding treatment planning, and monitoring healing. Several imaging modalities are commonly used during assessment.

Plain radiographs, including anteroposterior and lateral views, are standard in initial assessments and allow for fracture classification [6]. However, up to 20% of fractures, particularly occult fractures, may not be visible on initial X-rays [7].

Computed tomography (CT) offers superior visualisation of comminuted fractures and intra-articular involvement, aiding surgical planning [7]. Three-dimensional reconstruction can further enhance preoperative planning in complex periarticular fractures [8].

Magnetic resonance imaging (MRI) is useful in evaluating associated ligamentous or soft tissue injuries, particularly in periarticular fractures. However, MRI availability and cost remain significant barriers to its widespread use [8].

Angiography is indicated when vascular compromise is suspected, allowing timely intervention in cases of arterial injury. Doppler ultrasonography can serve as an effective initial screening tool and may reduce the number of unnecessary angiographic procedures [8].

Despite their benefits, imaging modalities have limitations. CT scans involve increased radiation exposure, and MRI is expensive and not always accessible in emergency settings. Moreover, reliance on imaging without appropriate correlation to clinical findings may result in inappropriate management decisions. Artificial intelligence-assisted image analysis has been explored to improve diagnostic accuracy, although further validation is necessary.

Multidisciplinary Approach to Assessment

The involvement of a multidisciplinary team is not just beneficial but crucial in enhancing the assessment and management of complex fractures. The combined expertise of orthopaedic surgeons, vascular surgeons, plastic surgeons, and rehabilitation specialists contributes significantly to optimising outcomes. The BOA/BAPRAS guidelines advocate for early collaboration in open fracture management to ensure adequate soft tissue coverage and minimise infection risks. Early involvement of rehabilitation specialists, including physiotherapists and occupational therapists, can aid in setting realistic functional goals and improving patient engagement in recovery.

Challenges and Limitations in Assessment

There is no simple algorithm that can be deployed that considers and weighs in all variables and can come up with a definitive plan. These complex limb injuries occur because of polytrauma, where each case presents its own unique challenges, modes of injury, injury types, and patient expectations.

Variables influencing the decision-making factors in salvage versus amputation for complex limb injuries

In the context of acute severe limb injuries, a decision must be made to either salvage the limb or proceed with a primary amputation. This decision remains a challenge of high significance to the trauma team and is usually a multidisciplinary decision. Some factors that affect decision-making are discussed below.

Haemodynamic Status

Haemodynamic status remains an important factor in deciding between salvage and amputation. These complex limb injuries often present in a polytrauma setting. The option of limb salvage should be considered only in patients who are haemodynamically stable and can tolerate the required surgical procedures, as well as the associated blood loss.

The dilemma in physiologically unstable patients is the systemic burden of multiple surgeries required for limb salvage. This must be carefully evaluated before deciding whether to proceed with amputation. The guiding principle of "life over limb" takes precedence in patients with persistent haemodynamic compromise. Ischaemic limbs do not pose an immediate threat to life compared to an exsanguinating limb, which must be addressed urgently [9].

Severity of Injury and Predictive Scoring Systems

One of the primary factors influencing the decision is the extent of bone and soft tissue injury. Scoring systems such as the Mangled Extremity Severity Score (MESS) have been developed to assist clinicians in evaluating the likelihood of successful salvage. MESS incorporates variables such as ischaemia time, shock, and the degree of skeletal and soft tissue damage. Studies have shown that a MESS score above 7 is predictive of poor salvage outcomes [10]. However, some studies have criticised MESS for its low predictive value in certain patient populations. Alternative scoring systems, such as the Ganga Hospital Open Injury Score (GHOIS), have been proposed to provide a more comprehensive assessment [11]. Critics argue that no single scoring system is sufficiently predictive to dictate treatment, as individual patient factors and surgeon expertise significantly influence outcomes [11].

Patient-Specific Assessment

Patient factors such as age, smoking status, diabetes, and osteoporosis influence fracture healing rates and the risk of complications. For example, smoking has been associated with a 30% increased risk of nonunion or delayed union in long bone fractures. The incorporation of frailty scores into assessment protocols may improve prognostic accuracy, particularly in elderly patients.

Functional outcomes: salvage versus amputation

Long-term functional outcomes are crucial when weighing salvage against amputation. The Lower Extremity Assessment Project (LEAP) multicentre prospective study found no significant difference in functional outcomes between patients undergoing salvage and those receiving amputations [12,13]. However, limb salvage is often associated with multiple surgical procedures, prolonged rehabilitation, and increased risk of complications such as osteomyelitis [12,13]. In contrast, early amputation may offer a faster return to function with prosthetic rehabilitation, particularly in cases with extensive soft tissue loss and neurovascular injury [12-14].

Infection Risk and Sepsis

Infection is a significant risk factor influencing the decision between salvage and amputation. Open fractures, particularly Gustilo-Anderson type III injuries, carry a high risk of deep infection and osteomyelitis [15]. Extensive soft tissue damage compromises vascularity and impairs immune responses, leading to increased susceptibility to infection [16]. Some studies have shown that infection rates in salvaged limbs can reach up to 40%, significantly delaying recovery and increasing morbidity [17]. Deep infections may lead to systemic sepsis, requiring aggressive management with prolonged antibiotic therapy and additional surgical interventions [18].

Amputation, in contrast, eliminates the source of infection in cases of severe contamination or necrotic tissue. Patients with persistent infections despite multiple debridements may benefit from early amputation, which can reduce hospitalisation time and improve survival rates [19]. However, it is important to consider the psychological and functional consequences of amputation before making a definitive decision.

Psychological and Quality of Life Considerations

Beyond physical function, psychological impact is a critical consideration. Studies indicate that depression, post-traumatic stress disorder (PTSD), and chronic pain are prevalent in patients with complex limb injuries, regardless of the treatment choice [20]. The LEAP study found that patients who underwent salvage often reported higher rates of depression and lower satisfaction with their outcomes compared to amputees. This suggests that while limb salvage is technically feasible, it may not always be in the patient's best psychological interest.

Economic and Healthcare Resource Implications

The financial burden of treatment also influences decision-making. Salvage procedures require multiple surgeries, long-term hospitalisation, and rehabilitation, increasing overall costs [21]. In contrast, amputation, though associated with higher initial expenses, may be more cost-effective in the long term due to a lower incidence of repeat procedures and hospitalisations. However, prosthetic costs and lifelong maintenance must also be considered [22].

Role of Multidisciplinary Teams in Decision-Making

A multidisciplinary approach is essential in managing complex limb injuries. Orthopaedic surgeons, vascular surgeons, plastic surgeons, rehabilitation specialists, and psychologists should be involved in the decision-making process [23]. Shared decision-making, incorporating patient preferences and values, ensures holistic management tailored to individual needs.

## Conclusions

Assessing and managing complex limb fractures requires a multifaceted approach integrating clinical evaluation, imaging, functional assessment, and multidisciplinary input. Both limb salvage and amputation have advantages and disadvantages. While limb salvage offers the potential for preserving the native limb, it is associated with prolonged recovery, higher complication rates, and often poorer psychological outcomes. Amputation, though initially distressing, can provide a more predictable functional recovery. The risk of infection and sepsis is a critical factor in the decision-making process, with limb salvage increasing the likelihood of deep infection and prolonged antibiotic use. The decision must be individualised, incorporating injury severity, functional prognosis, psychological impact, economic considerations, and infection risk. Future research should focus on refining predictive tools and enhancing rehabilitation strategies to optimise outcomes. Ultimately, a patient-centred approach that considers both medical and psychosocial factors is essential in managing these challenging cases.

## References

[1] Gustilo RB, Anderson JT (1976). Prevention of infection in the treatment of one thousand and twenty-five open fractures of long bones: retrospective and prospective analyses. J Bone Joint Surg Am.

[2] Court-Brown CM, Rimmer S, Prakash U, McQueen MM (1998). The epidemiology of open long bone fractures. Injury.

[3] American College of Surgeons (2018). ATLS: Advanced Trauma Life Support. Chicago: ACS.

[4] (2017). British Orthopaedic Association. Boast - open fractures. https://www.boa.ac.uk/resource/boast-4-pdf.html.

[5] (2017). British Orthopaedic Association. BOAST - diagnosis and management of compartment syndrome of the limbs. https://www.boa.ac.uk/resource/boast-10-pdf.html.

[6] Müller ME, Nazarian S, Koch P, Schatzker J (1990). The Comprehensive Classification of Fractures of Long Bones. https://link.springer.com/book/10.1007/978-3-642-61261-9.

[7] Corrales LA, Morshed S, Bhandari M, Miclau T 3rd (2008). Variability in the assessment of fracture-healing in orthopaedic trauma studies. J Bone Joint Surg Am.

[8] Tonetti J, Boudissa M, Kerschbaumer G, Seurat O (2020). Role of 3D intraoperative imaging in orthopedic and trauma surgery. Orthop Traumatol Surg Res.

[9] Beeharry MW, Walden-Smith T, Moqeem K (2022). Limb salvage vs. amputation: factors influencing the decision-making process and outcomes for mangled extremity injuries. Cureus.

[10] Johansen K, Daines M, Howey T, Helfet D, Hansen ST Jr (1990). Objective criteria accurately predict amputation following lower extremity trauma. J Trauma.

[11] Helfet DL, Howey T, Sanders R, Johansen K (1990). Limb salvage versus amputation. Preliminary results of the Mangled Extremity Severity Score. Clin Orthop Relat Res.

[12] Bosse MJ, MacKenzie EJ, Kellam JF (2002). An analysis of outcomes of reconstruction or amputation after leg-threatening injuries. N Engl J Med.

[13] MacKenzie EJ, Bosse MJ, Castillo RC (2004). Functional outcomes following trauma-related lower-extremity amputation. J Bone Joint Surg Am.

[14] Godina M (1986). Early microsurgical reconstruction of complex trauma of the extremities. Plast Reconstr Surg.

[15] Gustilo RB, Mendoza RM, Williams DN (1984). Problems in the management of type III (severe) open fractures: a new classification of type III open fractures. J Trauma.

[16] Pollak AN, McCarthy ML, Burgess AR (2000). Short-term wound complications after application of flaps for coverage of traumatic soft-tissue defects about the tibia. The Lower Extremity Assessment Project (LEAP) Study Group. J Bone Joint Surg Am.

[17] Patzakis MJ, Wilkins J (1989). Factors influencing infection rate in open fracture wounds. Clin Orthop Relat Res.

[18] Tetsworth K, Cierny G 3rd (1999). Osteomyelitis debridement techniques. Clin Orthop Relat Res.

[19] American Academy of Orthopaedic Surgeons (2019). American Academy of Orthopaedic Surgeons. Management of Orthopaedic Trauma: Surgical Site Infections Clinical Practice Guideline. Rosemont, IL: American Academy of. Management of Orthopaedic Trauma: Surgical Site Infections Clinical Practice Guideline.

[20] Doukas WC, Hayda RA, Frisch HM (2013). The Military Extremity Trauma Amputation/Limb Salvage (METALS) study: outcomes of amputation versus limb salvage following major lower-extremity trauma. J Bone Joint Surg Am.

[21] Chung KC, Saddawi-Konefka D, Haase SC, Kaul G (2009). A cost-utility analysis of amputation versus salvage for Gustilo type IIIB and IIIC open tibial fractures. Plast Reconstr Surg.

[22] Dillingham TR, Pezzin LE, MacKenzie EJ (2002). Limb amputation and limb deficiency: epidemiology and recent trends in the United States. South Med J.

[23] Gopal S, Majumder S, Batchelor AG, Knight SL, De Boer P, Smith RM (2000). Fix and flap: the radical orthopaedic and plastic treatment of severe open fractures of the tibia. J Bone Joint Surg Br.

